# Supplementary material for: From sequence to enzyme mechanism using multi-label machine learning
Source: BMC Bioinformatics. 2014 May 19;15:150. doi: 10.1186/1471-2105-15-150 (PMC4229970; doi:10.1186/1471-2105-15-150)
Supplement: Additional file 2 — Java code of ml2db. Additional file ml2db_code.tar.gz contains the Java source code to run the multi-label machine learning experiments and save the results to database. The code’s Javadoc is included. [file 1471-2105-15-150-S2.zip › additional file 2/ml2db/ml2db/doc/mulan/core/Util.html]

Util


JavaScript is disabled on your browser.


- Overview
- Package
- Class
- Use
- Tree
- Deprecated
- Index
- Help

- Prev Class
- Next Class

- Frames
- No Frames

- All Classes

- Summary:
- Nested |
- Field |
- Constr |
- Method

- Detail:
- Field |
- Constr |
- Method


mulan.core

## Class Util

- java.lang.Object
- - mulan.core.Util

- ---

    

  ```
  public class Util
  extends java.lang.Object
  ```

  Class which provides various utility methods.

  Author:
  :   Jozef Vilcek

- - ### Constructor Summary

    Constructors

    Constructor and Description

    `Util()`
  - ### Method Summary

    Methods

    | Modifier and Type | Method and Description |
    | --- | --- |
    | `static java.lang.String` | `getNewLineSeparator()` Returns a correct new line separator string for the underlying operating system. |
    | `static int` | `RandomIndexOfMax(double[] array, java.util.Random rand)` Procedure to find index of maximum value in the specified array. |

    - ### Methods inherited from class java.lang.Object

      `equals, getClass, hashCode, notify, notifyAll, toString, wait, wait, wait`

- - ### Constructor Detail


    - #### Util

      ```
      public Util()
      ```
  - ### Method Detail


    - #### RandomIndexOfMax

      ```
      public static int RandomIndexOfMax(double[] array,
                         java.util.Random rand)
      ```

      Procedure to find index of maximum value in the specified array.
      If there is only one unique maximum, the index of this maximum is returned.
      If there are more values equal to the maximum, one of these values is randomly
      selected and its index is returned.

      Parameters:
      :   `array` - the array in which the maximum value should be find
      :   `rand` - random instance used to select the value if more values equal
          to the maximum are present in the array

      Returns:
      :   the index of find maximum value


    - #### getNewLineSeparator

      ```
      public static java.lang.String getNewLineSeparator()
      ```

      Returns a correct new line separator string for the underlying operating system.

      Returns:
      :   the new line separator string


- Overview
- Package
- Class
- Use
- Tree
- Deprecated
- Index
- Help

- Prev Class
- Next Class

- Frames
- No Frames

- All Classes

- Summary:
- Nested |
- Field |
- Constr |
- Method

- Detail:
- Field |
- Constr |
- Method
